# Supplementary material for: Polyketide synthase gene clusters in the ericoid mycorrhizal fungus Oidiodendron maius: genome-wide prediction, expression profiling, and functional characterization of knockout mutants
Source: Front Plant Sci. 2026 Sep 16;17:1924430. doi: 10.3389/fpls.2026.1924430 (PMC13623903; doi:10.3389/fpls.2026.1924430)
Supplement: Supplementary file 1 [file SupplementaryFile1.zip › Supplementary Materials/Supplementary Folder 1.zip!/Oidma1_AssemblyScaffolds_Repeatmasked/knownclusterblast/cluster8/jgi.p_Oidma1_66911_mibig_hits.html]

| MIBiG Protein | Description | MIBiG Cluster | MiBiG Product | % ID | % Coverage | BLAST Score | E-value |
| --- | --- | --- | --- | --- | --- | --- | --- |
| CAE17550.1 | oxygenase | BGC0000210 | Chromomycin\_A3\_biosynthetic\_gene\_cluster | 27 | 101.857585139 | 102 | 6.8e-22 |
| ABB88524.1 | Baeyer-Villiger\_monooxygenase | BGC0000050 | E-837\_biosynthetic\_gene\_cluster | 28 | 101.238390093 | 102 | 6.8e-22 |
| CBW45655.1 | putative\_oxygenase | BGC0000952 | Pristinamycin\_biosynthetic\_gene\_cluster | 27 | 103.405572755 | 101 | 1.5e-21 |
| CAE17536.1 | oxygenase | BGC0000210 | Chromomycin\_A3\_biosynthetic\_gene\_cluster | 30 | 98.4520123839 | 101 | 1.5e-21 |
| CAG14963.1 | oxygenase | BGC0000253 | Oviedomycin\_biosynthetic\_gene\_cluster | 28 | 104.643962848 | 97 | 2.8e-20 |
| ACR50781.1 | putative\_FAD-dependent\_oxygenase | BGC0000163 | Tetronasin\_biosynthetic\_gene\_cluster | 27 | 102.786377709 | 95 | 1.4e-19 |
| CAA07757.1 | oxygenase | BGC0000246 | Mithramycin\_biosynthetic\_gene\_cluster | 25 | 97.213622291 | 90 | 3.5e-18 |
| CAK50777.1 | oxygenase | BGC0000247 | Mithramycin\_biosynthetic\_gene\_cluster | 25 | 97.213622291 | 90 | 3.5e-18 |
| AHZ61858.1 | Lom24 | BGC0000240 | Lomaiviticin\_biosynthetic\_gene\_cluster | 26 | 98.1424148607 | 89 | 7.7e-18 |
| AGZ15446.1 | putative\_polyketide\_oxygenase/hydroxylase | BGC0001036 | Polyoxypeptin\_biosynthetic\_gene\_cluster | 28 | 101.857585139 | 87 | 3.8e-17 |
| AAL33754.1 | putative\_monooxygenase/hydroxylase | BGC0000421 | Safracin\_biosynthetic\_gene\_cluster | 27 | 103.095975232 | 86 | 6.5e-17 |
| AEE65462.1 | oxygenase | BGC0000223 | Fluostatin\_biosynthetic\_gene\_cluster | 28 | 99.3808049536 | 86 | 6.5e-17 |
| ACY06308.1 | FAD-dependent\_monooxygenase | BGC0001042 | Sanglifehrin\_A\_biosynthetic\_gene\_cluster | 24 | 100.0 | 80 | 3.6e-15 |
| AGD80608.1 | monooxygenase | BGC0000394 | Naphthyridinomycin\_biosynthetic\_gene\_cluster | 26 | 102.476780186 | 77 | 2.3e-14 |
| AHZ61861.1 | Lom27 | BGC0000240 | Lomaiviticin\_biosynthetic\_gene\_cluster | 25 | 96.5944272446 | 77 | 2.3e-14 |
| CAK50794.2 | oxygenase | BGC0000247 | Mithramycin\_biosynthetic\_gene\_cluster | 27 | 100.309597523 | 77 | 3e-14 |
| ABI22123.1 | monooxygenase | BGC0000422 | Saframycin\_A\_biosynthetic\_gene\_cluster | 24 | 105.882352941 | 74 | 3.4e-13 |
| ABP54650.1 | monooxygenase,\_FAD-binding | BGC0000241 | Lomaiviticin\_biosynthetic\_gene\_cluster | 25 | 98.1424148607 | 72 | 9.8e-13 |
| ABP54636.1 | monooxygenase,\_FAD-binding | BGC0000241 | Lomaiviticin\_biosynthetic\_gene\_cluster | 26 | 77.7089783282 | 71 | 1.7e-12 |
| AHZ61847.1 | Lom13 | BGC0000240 | Lomaiviticin\_biosynthetic\_gene\_cluster | 26 | 77.7089783282 | 71 | 2.2e-12 |
| CAG14970.1 | oxygenase | BGC0000253 | Oviedomycin\_biosynthetic\_gene\_cluster | 26 | 100.619195046 | 69 | 6.3e-12 |
| AAG30200.1 | oxygenase | BGC0000261 | R1128\_biosynthetic\_gene\_cluster | 24 | 106.811145511 | 69 | 1.1e-11 |
| ABP54647.1 | monooxygenase,\_FAD-binding | BGC0000241 | Lomaiviticin\_biosynthetic\_gene\_cluster | 29 | 53.8699690402 | 66 | 7e-11 |
| ACN64854.1 | PokO4 | BGC0001061 | Polyketomycin\_biosynthetic\_gene\_cluster | 28 | 56.0371517028 | 65 | 1.6e-10 |
| AKD43495.1 | Monooxygenase | BGC0001409 | Dutomycin\_biosynthetic\_gene\_cluster | 27 | 56.0371517028 | 64 | 3.5e-10 |
| BAJ07857.1 | putative\_oxygenase | BGC0000232 | Hatomarubigin\_biosynthetic\_gene\_cluster | 30 | 56.6563467492 | 62 | 7.7e-10 |
| CAA60567.1 | putative\_oxygenase | BGC0000276\_c4 | Urdamycin\_biosynthetic\_gene\_cluster | 27 | 53.560371517 | 60 | 3.8e-09 |
| AFU65887.1 | DacO3 | BGC0000216 | Dactylocycline\_biosynthetic\_gene\_cluster | 27 | 54.7987616099 | 54 | 2.1e-07 |
| AEI98657.1 | CtcN | BGC0000209 | Chlortetracycline\_biosynthetic\_gene\_cluster | 26 | 52.3219814241 | 52 | 1e-06 |
| ACP19351.1 | SaqE | BGC0000267 | Saquayamycin\_Z\_/\_galtamycin\_B\_biosynthetic\_gene\_cluster | 24 | 55.1083591331 | 49 | 6.8e-06 |
